# Customised Intrusion Detection for an Industrial IoT Heterogeneous Network Based on Machine Learning Algorithms Called FTL-CID

**DOI:** 10.3390/s23010321

**Published:** 2022-12-28

**Authors:** Nasr Abosata, Saba Al-Rubaye, Gokhan Inalhan

**Affiliations:** School of Aerospace, Transport and Manufacturing, Cranfield University, Cranfield MK43 0AL, UK

**Keywords:** Internet of Things (IoT), security, distributed sensors, intrusion detection, machine learning, application, AMI, attacks

## Abstract

Technological breakthroughs in the Internet of Things (IoT) easily promote smart lives for humans by connecting everything through the Internet. The de facto standardised IoT routing strategy is the routing protocol for low-power and lossy networks (RPL), which is applied in various heterogeneous IoT applications. Hence, the increase in reliance on the IoT requires focus on the security of the RPL protocol. The top defence layer is an intrusion detection system (IDS), and the heterogeneous characteristics of the IoT and variety of novel intrusions make the design of the RPL IDS significantly complex. Most existing IDS solutions are unified models and cannot detect novel RPL intrusions. Therefore, the RPL requires a customised global attack knowledge-based IDS model to identify both existing and novel intrusions in order to enhance its security. Federated transfer learning (FTL) is a trending topic that paves the way to designing a customised RPL-IoT IDS security model in a heterogeneous IoT environment. In this paper, we propose a federated-transfer-learning-assisted customised distributed IDS (FT-CID) model to detect RPL intrusion in a heterogeneous IoT. The design process of FT-CID includes three steps: dataset collection, FTL-assisted edge IDS learning, and intrusion detection. Initially, the central server initialises the FT-CID with a predefined learning model and observes the unique features of different RPL-IoTs to construct a local model. The experimental model generates an RPL-IIoT dataset with normal and abnormal traffic through simulation on the Contiki-NG OS. Secondly, the edge IDSs are trained using the local parameters and the globally shared parameters generated by the central server through federation and aggregation of different local parameters of various edges. Hence, transfer learning is exploited to update the server’s and edges’ local and global parameters based on relational knowledge. It also builds and customised IDS model with partial retraining through local learning based on globally shared server knowledge. Finally, the customised IDS in the FT-CID model enforces the detection of intrusions in heterogeneous IoT networks. Moreover, the FT-CID model accomplishes high RPL security by implicitly utilising the local and global parameters of different IoTs with the assistance of FTL. The FT-CID detects RPL intrusions with an accuracy of 85.52% in tests on a heterogeneous IoT network.

## 1. Introduction

The Internet of Things (IoT) is a collection of smart devices, such as mobile phones, smart sensors, cameras, and laptops that are connected through the Internet [1]. Various technological breakthroughs in the industrial IoT (IIoT) have recently made numerous applications possible, ranging from small industries to smart cities, which have become integral to several aspects of human life [2]. The routing protocol for low-power and lossy networks (RPL) is a standardised IIoT routing strategy. It is well suited to establishing communication infrastructure among tiny, resource-constrained IIoT devices. However, the resource-limited nature of IoT devices and critical smart infrastructures motivate hackers to launch various attacks within the RPL-IIoT scenario, and RPL security is paramount. A common method of RPL-IIoT attack detection is to employ a machine learning (ML)-based intrusion detection system (IDS) [3,4]. However, most traditional ML-based IDS solutions are centralised and do not have the flexibility to detect the variety and full range of RPL attacks on IIoT applications [5]. Heterogeneous IIoT networks perform various network functions and present a challenge to intrusion detection, as there are various data and attack types. ML-based solutions are typically evaluated with small datasets, resulting in a lack of detection of novel attacks in real time. The RPL is mainly utilised in heterogeneous IIoT environments, and it is crucial to evaluate the IDS security model with a variety of IIoT intrusions presented in diverse RPL-IIoT networks. Most federated learning-assisted IDS models solve intrusion detection problems in heterogeneous IoT environments, but there are no specified FTL-based IDS models for heterogeneous RPL-IoT environments.

IDS plays a crucial role in ensuring the security and confidentiality of such devices. ML with systems for intrusion detection has gained significant traction due to its high classification precision. FL provides collaborative learning among devices without requiring data sharing with a centralised server. Owing to its characteristics, ML can be taught across various devices and servers with decentralised data over multiple iterations.

Federated learning (FL)-based IDS models are currently effective for IIoT intrusion detection. They can train a deep learning models with global knowledge and ensure high security while protecting user data [6]. Depending on the data sample and feature distribution, FL is categorised into three types: horizontal FL, vertical FL, and federated transfer learning (FTL) [7,8]. FL-based IDS models enhance learning efficiency with reduced overhead and protect user information by achieving distributed learning at the edges. However, the insufficiency of unique data labelling and the dissimilarity of data features make FL-based IDS systems challenging. Transfer learning (TL) solves such issues by transferring the abundant labelled source domain knowledge to the edges. This avoids the lengthy learning time of particularised RPL-IIoT for designated applications. Moreover, an FLT algorithm is a novel concept that achieves an effective learning model by implicitly integrating the local and global knowledge of the IIoT network. 

In this paper, we present an FTL-assisted customised IDS model, the FT-CID, to detect intrusions in a heterogeneous RPL-IIoT network. The main contributions of the FT-CID are as follows.

- To the best of our knowledge, the FT-CID is the first model to apply FTL to a heterogeneous RPL-IIoT IDS security model. This approach aggregates the data from the IIoT application domain. It accomplishes a customised attack detection model with global knowledge for the IIoT network by transferring and federating learning knowledge between the server and edges.
- First, the FT-CID uses FL to accumulate the local learning models of different RPL-IIoT networks and generate a global shared parameter for edges to detect attack types of existing and unknown intrusions.
- Secondly, using relational knowledge transfer learning, the FT-CID achieves a personalised IDS by efficiently transferring distributed local and global parameters between the server and IIoT network or sensors with high security.
- Thirdly, the FT-CID detects and classifies intrusions under multiple classes using the customised IDS model and ensures a rich level of routing security for diverse IIoT scenarios without data leakage.
- Finally, the FT-CID reflects the security of a heterogeneous IIoT environment by conducting an experimental evaluation of the IIoT dataset: advanced metering infrastructure (AMI).

The remainder of this paper is organised as follows. Section 2 presents the related work, and Section 3 outlines the network architecture, system model, and attack model. Section 4 presents the proposed FT-CID design process with appropriate figures, equations, and algorithms. Section 5 shows the experimental setup and evaluation of FT-CID. Section 6 presents the experimental results. Finally, Section 7 summarises this paper.

## 2. Related Work

In recent decades, several intrusion detections studies have employed machine learning models to defend the IoT network from the attacks and threats studied in [9]. The model proposed in [10] detects automated industrial IoT attacks using a machine learning algorithm. It performs functional shape analysis to extract and represent features, enhancing the machine learning algorithm’s performance during attack detection. By modelling waveforms through functional representation for feature extraction, it detects different types of IIoT attacks through a novel early detection strategy. The authors of [11] presented a novel supervised machine-learning-based IDS strategy to identify security attacks in RPL-based cyber security systems. However, conventional machine-learning-based solutions mainly focus on detecting the minimum number of attacks, and they fail to cover most attacks that occur in the RPL-IoT scenario. They are also evaluated with simple IoT datasets, which could be more effective in reflecting IoT heterogeneity. The model presented in [12] utilises a machine learning model to identify combined attacks in the IoT environment. It detects attacks against two main RPL objective functions, i.e., minimum rank with hysteresis objective function (MRHOF) and objective function zero (OF0). Such a model constructs a novel IoT dataset with optimal IoT features and attacks to efficiently detect attacks. The authors of [13] presented a DETector of rOutiNg Attacks in RPL (DETONAR) model that combines the advantages of anomaly- and signature-based IDS models to successfully detect malicious behaviour in network traffic. To handle the issue of a lack of datasets of the RPL-IoT, DETONAR introduces a routing attacks dataset for RPL (RADAR) in which more than fourteen RPL attacks are covered. However, most of the abovementioned solutions exploit a centralised means of detecting attacks, whereas the RPL-IoT is mostly distributed among many regions. Hence, it is crucial to design a novel machine-learning-based IDS solution to effectively handle IoT heterogeneity. Federated learning is a distributed learning model that learns heterogeneous IoT data by enabling various IoT services, as investigated in [14]. To provide security for IoT networks, the network intrusion detection model [15] adopts the federated learning model with joint training by deep learning to improve the accuracy of intrusion detection and protect privacy in network traffic.

Furthermore, several IoT studies have employed a federated learning model to preserve privacy [16,17,18]. The authors of [16] proposed a supportive twin delayed DDPG (S-TD3) algorithm to authenticate and preserve the privacy of the IIoT network with the assistance of the federated transfer learning model. In addition to ensuring the privacy and security of federated transfer learning blockchains, transfer-based blockchains with an authentication mechanism enrich the security and preservation standards for IIoT applications. The IoT anomaly detection model [17] adopts federated learning with the potential advantage of privacy preservation and the design of a blockchain-based asynchronous and decentralised federated learning model. Consequently, it avoids single-point failure and ensures data integrity with improved efficiency during anomaly detection. Moreover, it applies generative adversarial networks to design a private federated learning model to optimise data utility during the training process. It preserves privacy in the decentralised IoT anomaly detection system. The IoT privacy-preserving framework [18] detects malware without sharing sensitive data through the federated learning model’s training and evaluation of both the supervised and unsupervised models. It supports the privacy preservation of IoT devices such as 5G, Wi-Fi, or B5G networks during computation offloading and processing in IoT devices. The work in [19] shows that FL-based IDS models are susceptible to backdoor attacks on the IoT. To discuss and rectify such a problem, it presents a novel data poisoning attack in which a malicious intruder implants a backdoor of aggregated information in the detection model, leading to incorrect classification of malicious and benign information. Thus, the malicious intruder can gradually poison the detection strategy by only exploiting the compromised IoT devices to inject an amount of malicious traffic. Moreover, it motivates researchers to innovate novel FL IDS models for the IoT to optimise performance. The authors of [20] introduced a defence model of FL against gradient-based reconstruction attacks in resource-limited IoT environments. Such a model compares analyses of the loss of the training model and designs an adaptive communication mechanism by adjusting the frequency and parameter compression, resulting in a high-security achievement. The framework presented in [21] advocates a personalised federated learning model to investigate the heterogeneous issues associated with the IoT environment. It also accomplishes personalisation and maximises the performance of intelligent IoT devices by integrating four different federated learning methods: federated transfer learning, federated meta-learning, federated multitask learning, and federated distillation. The federated machine-learning-based IDS model proposed in [22] trains the models of IoT devices based on local data and maintains the security of sensitive IoT information. Furthermore, federated-learning-based server aggregation with local models maximises the detection efficiency of clients. The authors of [23] proposed a federated-learning-based attention-gated recurrent unit (FedAGRU) model to assure security at the edges of wireless networks. FedAGRU updates ubiquitous learning without sharing clients’ basic information to the server and optimises the learning model. Furthermore, it neglects unimportant parameter transmissions to the server with the help of an attention mechanism and greatly enhances the security performance with minimum communication overhead. The authors of [24] presented a federated-learning-assisted intelligent IDS model that exploits the FedACNN algorithm to detect intrusions in edge-enabled industrial IoT environments. It solves the issues associated with centralised machine learning models by transferring model parameters to the global server with high security instead of sending raw data. It considerably improves IDS detection accuracy by achieving an accelerated global model using the federated learning concept. The authors of [25] proposed an ensemble multiview federated-learning-based intrusion detection model called MV-FLID. It views IoT data traces differently and forms an effective decentralised learning process with the help of federated learning. Furthermore, MV-FLID identifies and classifies intrusions and provides strong security against attacks. It improves learning efficiency by viewing the data of diverse attack classes in multiple aspects. The authors of [26] presented a three-layer FL architecture for an industrial IoT environment. In this model, the gateways are locally trained with a deep neural network (DNN) model and optimise the learning process by globally federating the locally trained models. Transfer learning models have recently attracted significant attention in detecting intrusions in RPL-based resource-constrained IoT networks by transferring knowledge from the source domain to the target domain. Another study [27] investigated the role of transfer learning in transferring knowledge for intrusion detection for new devices and transferring knowledge to detect new attacks. The model proposed in [28] uses the advantages of federated and transfer learning to improve the generalisation training efficiency of the detection system in an industrial IoT environment. With the integration of edge learning in the IIoT network to resolve resource-constrained IIoT devices, securing data transmission with the blockchain becomes crucial, along with the transfer learning model for the federated learning environment, to increase the versatility and efficiency of the training model. As a collaborative learning strategy to handle IoT device training issues with a large dataset, deep transferring models were used in [29] for industrial IoT environments. The authors of [30] presented a deep-transfer-learning-based traffic classification model for the 5G IoT environment. Such a model finetunes the classification model with a small set of labelled data by extracting the abundant labels of multiple domains. To denoise the raw ECG signal of patients in IoT healthcare, the model proposed in [31] utilises a federated-learning-assisted CNN-based autoencoder. Similarly, the authors of [32] proposed a federated-transfer-learning-based IDS model for the Internet of Medical Things environment. Such a model performs unique reconstruction with raw input and provides unique data to the federation process, resulting in improved healthcare IoT performance. It runs the classification algorithm on edge devices to handle the issue of network resource scarcity. However, most federated-learning-assisted IDS models solve the intrusion detection issues of heterogeneous IoT environments, whereas there are no specified FTL-based IDS models for heterogeneous RPL-IoT environments. 

## 3. System Model

**Figure 1:**
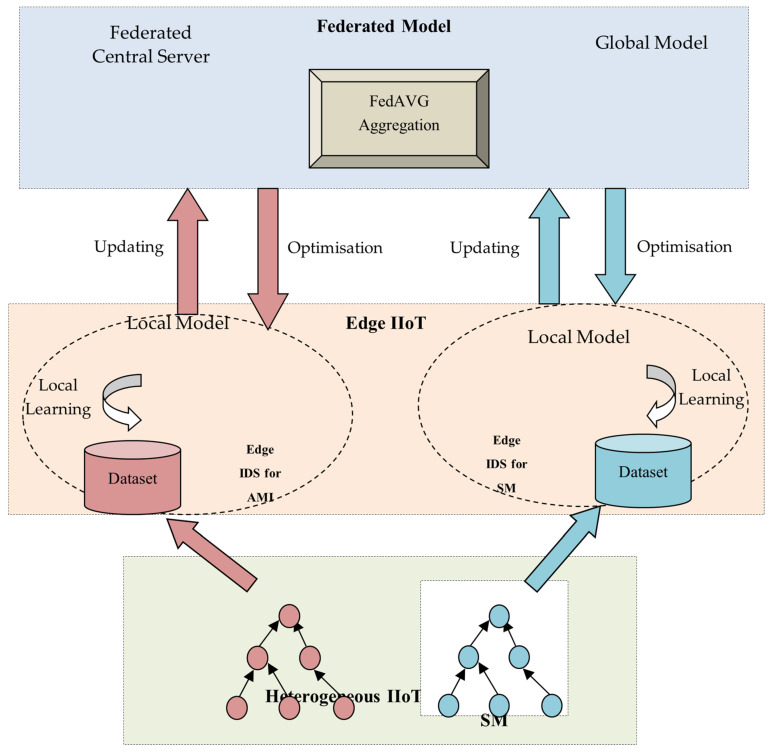
Architecture of heterogeneous IIoT network.

The architecture of the proposed FT-CID is shown in Figure 1. The FT-CID model design assists in intrusion detection for heterogeneous IIoT networks. To demonstrate the process of the FT-CID, IIoT networks are modelled: IIoT1 = advanced metering infrastructure (AMI). The edge devices of the corresponding IIoT network are denoted as E-AMI. First, the edge devices collect data from different IIoT devices through the RPL routing process. The FT-CID then instructs ‘N’ different edges of the IIoT network to obtain traffic data and construct the datasets.

An IIoT network refers to a heterogeneous network that consists of a set of IoT devices, such as smartphones and smart sensors, cameras, and meters. The FT-CID comprises the following aspects: IIoT devices, edges of the IIoT network or sensors, and a central server. The devices in an IIoT network are tiny and have constrained energy, storage space, and bandwidth. Compared with resource-constrained IIoT devices, edge nodes have enormous resource capabilities. Therefore, the FT-CID places a signature-based IDS on the edge of the IIoT networks. Unlike resource-limited IIoT devices and edges, such as smart watches, fire alarms, security systems, and medical sensors, the central server has enormous processing and storage capability. In FL, the central or global server gains knowledge from a heterogeneous IIoT network instead of storing or processing the data that belong to the sensors and thus facilitates a distributed IDS at the edge of those networks. The IIoT network considered in the FT-CID is represented as a set of heterogeneous IIoT (HIIoT) = {IIoT1}, where IIoT1 = AMI. The edges of the IIoT network are denoted by {E-AMI}, and the traffic data collected from the heterogeneous IIoT network are represented by {D1}. By building a local learning model, the edge devices are responsible for learning the patterns from the local dataset gathered from the distributed IIoT network. The central server exploits a traditional dataset to construct a predefined global learning model. It partially retrains the global learning model with the assistance of the local model parameters provided through the TL process. The data collected from the heterogeneous IIoT network comprises normal and abnormal data. Finally, a convolutional neural network (CNN)-based learning model in the FT-CID classifies the data at the edges as normal or abnormal based on the global learning parameters provided by the central server.

Attack Model: The RPL is exploited in heterogeneous IIoT environments, and the attacks faced in such environments are also different. To experiment with the intrusion detection model, we built a dataset with a design of intrusion patterns from RPL protocol-related attacks, such as rank, version, hello flood, denial of service (DoS), blackhole, Sybil, selective forwarding, sinkhole, theft, and wormhole attacks. In the FT-CID, all the attacks mentioned above are modelled as intrusions, which addresses the binary classification problem of having only normal and intrusion samples. In a blackhole attack, a malicious intruder may attempt to throw packets away instead of forwarding them. A sinkhole attack is performed by changing the RPL ranks and obtaining a large amount of traffic from the network to alter its RPL topology. In a rank attack, a malicious intruder disseminates false ranks to enjoy the network benefits. Wormhole attackers employ the off-band communication of two different entities to perform attacks. During hello flooding, the malicious node exploits DODAG information object (DIO) messages of destination-oriented directed acyclic graphs (DODOG) to launch attack activity. A clone ID attacker mimics legitimate nodes to diminish the network performance. Traffic analysis attackers exploit the features and data patterns of communication and launch harmful activities in a network. During a sniffing attack, a malicious node notices the network traffic exchanges between two entities and performs attacking activities. Hence, an efficient learning model is crucial to identify intrusions across normal samples. 

Learning Model: An FL model contains a single server and multiple sensors, in which the server refers to the remote cloud server and the sensors refer to the IIoT network. The main objective of FL is to enable a range of knowledge to be gained from multiple IoT networks by utilising the parameters of the learning model instead of directly storing and processing all data of the distributed sensors on a central server. In an FL model, the edge of the IIoT network independently trains the local datasets generated from the heterogeneous IIoT application with the deep learning model. The federated model then provides the local learning model with parameters as the input to the global server through the edge network. The remote server provides a global shared model with the updated parameters in the FL. To model intrusion detection for an IIoT network, FTL is employed to establish communication across the IoT networks and obtain the different intrusion patterns of the local training data distributed across different silos and samples. Moreover, to address the issue of learning from a small number of attack behaviours in the IIoT network, we modelled FTL for RPL-IIoT intrusion detection. A CNN model is adopted as the FTL’s local and global learning model with two different transfer scenarios: parameter transfer and relational knowledge transfer. Finally, the improved CNN model detects intrusions in heterogeneous IIoT networks. 

## 4. Proposed System Design Overview

**Figure 2:**
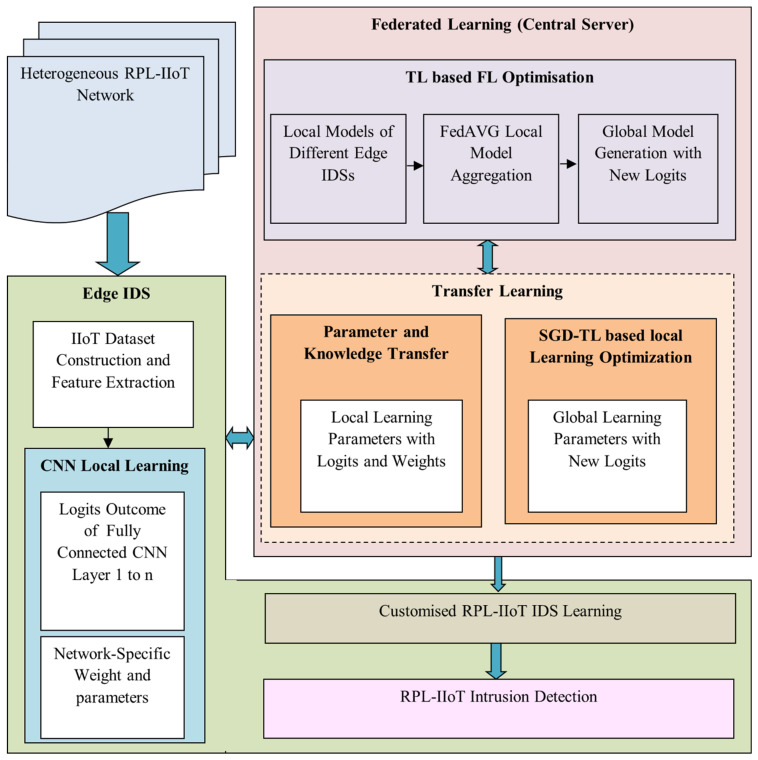
Overview of the design of the proposed FT-CID model.

The aim of this work is to design a novel RPL-IIoT security protocol with intrusion detection and introduce an optimised FTL-assisted customised IDS model: the FT-CID. The proposed FT-CID model is intended to achieve a very high intrusion detection accuracy in heterogeneous RPL-IIoT networks by adopting an FTL approach. The FTL model shares global attack knowledge among the various IIoT application scenarios generated based on the unique local learning parameters oof a distributed IDS model. A design overview of the FT-CID is shown in Figure 2. The FT-CID design process can be explained according to three steps. First, the centralised server initiates the network and sends a predefined learning model to the edges to trigger the local learning process on the data. The edges collect unique IIoT application data from application-specific IIoT networks and generate a local copy for each IIoT using a CNN classifier. Thirdly, the optimised FTL-based customised IDS model detects various RPL-IIoT intrusions based on local learning data and is optimised using a global learning model. Instead of transferring raw input data or results from the local learning model, the FT-CID transfers only the non-normalised prediction output as the logit outcome of a fully connected CNN with an IIoT network or application-specific weight values to optimise the global model generation. After receiving the logits from the local learning model, the centralised server initiates federated aggregation on local model logits, considering the degree of importance in the weights of local learning model parameters and logits for the IIoT network. The FT-CID model can then generate novel logit-based global shared parameters, such as the number of hidden layers and neurons and activation functions, to optimise the local learning model of the different RPL edge devices. Inspired by the idea of an attention mechanism [25], the FT-CID minimises the number of communications rounds and maximises learning convergence. With the federation of different local RPL-IIoT network parameters, the FT-CID achieves strong security and generalisation attack detection ability in heterogeneous IIoT networks. Finally, the central server sends back the globally shared learning parameters by utilising TL for the IIoT network to build customised learning models. Hence, the FT-CID model focuses on stochastic gradient descent (SGD)-based TL optimisation to improve the learning accuracy of each edge IDS without increasing the training time, which is repeated until the learning process converges. Intrusions are identified based on the global federated and unique attack knowledge acquired during testing. Thus, the distributed-learning-based detection model overcomes the shortcomings of centralised intrusion detection models and improves RPL security compared to a heterogeneous IIoT network without degrading network efficiency.

**Figure 3:**
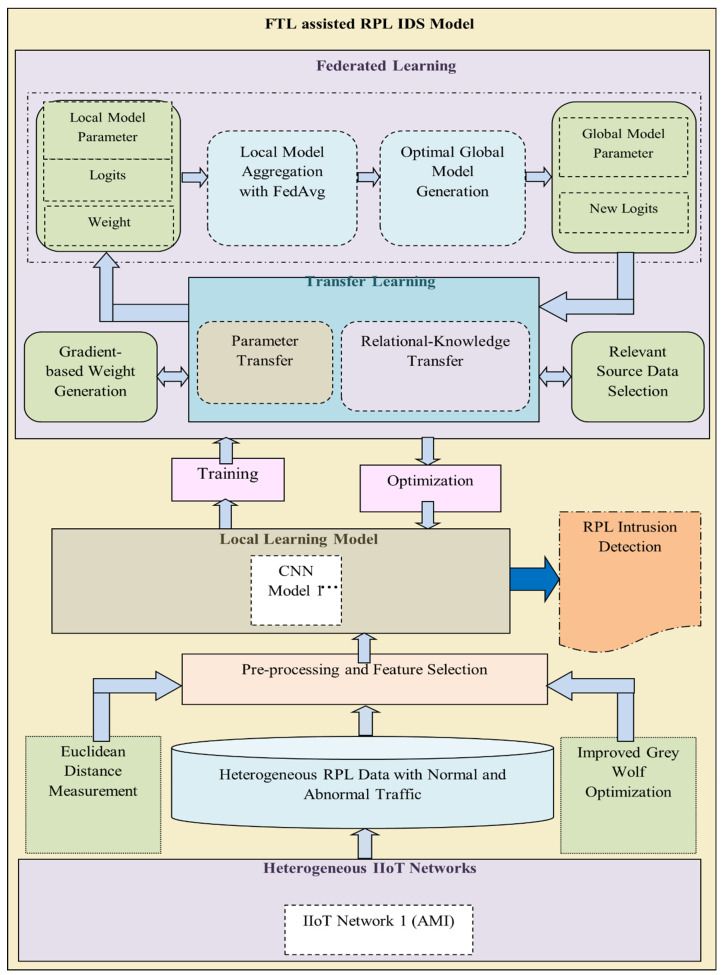
Process flow of the proposed FT-CID model.

Figure 3 shows the process involved in the proposed FT-CID model. First, the FT-CID model collects RPL data traffic with normal intrusion samples from the heterogeneous IIoT networks. The model preprocesses the collected traffic samples using Euclidean distance measurement to remove noisy data and then selects potential features from the preprocessed data from the IIoT network with the assistance of the improved grey wolf optimisation (IGWO) algorithm. The model then adopts the CNN model for local learning and generates logits for a local dataset of the IIoT network. The FT-CID model uses TL to communicate between the sensor’s local learning model and the server’s global learning model. After training the local model at the edge of the IIoT networks, the FT-CID model utilises the TL model with parameter transfer and relational knowledge transfer to update the global model. In the FT-CID, the TL model is associated with gradient-based weight generation for each local learning model and transfers the local learning model parameters, logits, and weights to the global learning model with the help of federated averaging (FedAVG)-based aggregation. Because the global model is trained using the transferred parameters, the FT-CID model optimises each local learning model by transferring knowledge, including the global parameters and logits, through TL in association with parameter transfer and knowledge transfer. Moreover, it selects the relevant source data to train the TL model with respect to the target domain of the IIoT network and thus optimises the local learning model with the assistance of the SGD model to detect intrusion samples in the heterogeneous IIoT networks. 

### 4.1. Dataset Construction

The RPL-IIoT has a distributed and hierarchical system model that necessitates personalised and distributed security solutions instead of a centralised security model. Hence, the proposed FT-CID model relies heavily on the RPL data collected from the IIoT application domain to build a distributed security protocol. Due to the ethical, technical, and economic complications of modelling a real IIoT network infrastructure with real-time operation, building or generating simulation datasets is vital for enabling intrusion detection in an IIoT environment [33,34]. Hence, in this study, we designed an IIoT network architecture through simulation on the Contiki-NG OS. The Cooja environment emulates an IIoT network with real CPU and memory power as the host workstation in the simulation design to generate normal and intrusion samples. The simulation design of the IIoT network is based on the considerations of portability and applicability in a real-world scenario. The data collection process is no different than a heterogeneous IIoT network. It generates the same feature space because the hardware and protocols used by all the nodes in the IIoT network remain the same. In the FT-CID model, the data are collected from the IIoT network by involving AMI. However, as the FT-CID builds IIoT datasets with various data samples and similar features, this may result in redundant and non-informative data. Inputting the collected data directly into the learning process escalates the time and maximises the algorithm complexity. Hence, to ensure quality decision making and reduce training time and algorithm complexity, it is crucial to preprocess the data and select optimal feature subsets in the IDS.

### 4.2. Preprocessing and Feature Selection

As a result of the enormous amount of erroneous and redundant data in the collected IIoT datasets, removing such data from the datasets improves the accuracy of the IDS and reduces the burden on the IDS learning process. First, the FT-CID model applies Euclidean distance measurement to the raw dataset to remove erroneous and redundant data. The FT-CID model then employs IGWO on the dataset $\left(D_{\mathrm{HIIoT}}\right)$ to select the optimal feature subsets for the IIoT network [35]. IGWO is a type of swarm intelligence algorithm in which optimisation is based on the three best solutions for food searching and hunting behaviours. According to the IGWO algorithm, the proposed FT-CID model obtains the best solutions for three wolves from the preprocessed data based on hunting behaviour using Equation (1).

(1) $$\left(t+1\right)=\frac{\left(x_{\alpha }-A_{1}\;*\;\left|C_{1}{\;*\;X}_{\alpha }-X\right|\right)\left(x_{\beta }-A_{2}\;*\;\left|C_{2}{\;*\;X}_{\beta }-X\right|\right)\left(x_{\delta }-A_{3}\;*\;\left|C_{3}{\;*\;X}_{\delta }-X\right|\right)}{3}$$

where $A_{1},A_{2},{\;A}_{3},C_{1},C_{2},{\;\mathrm{and}\;C}_{3}$ are all random vectors, and α, β, and δ are the best, second-best, and third-best positions of the three wolves, respectively. In every round, δ represents the worst positions of neglected features. Finally, the FT-CID model selects the optimal feature. The selected subset is based on the values of search optimisers and delta. The selected feature subset is provided as the input to the CNN model of IIoT1. Furthermore, it repeats the IGWO-based feature selection process on the entire heterogeneous IIoT network to enforce a network sharing process of the deep learning model on the local dataset, i.e., AMI. 

### 4.3. Optimised FTL-assisted Edge IDS Learning 

Due to privacy constraints, training the learning model with an enormous amount of centralised data is impossible in distributed IIoT domains. Although FL-based models effectively address the privacy constraint, an inadequate number of standard datasets and a lack of consideration of the unique characteristics of the IIoT lead to inaccurate intrusion detection. Hence, the FT-CID model integrates the concept of TL with FL to build accurate IDS models with minimum learning time, with the advantage of utilising the learned knowledge of a source domain in various target domains in the TL model. The FT-CID model adopts FTL to resolve the security issues associated with a heterogeneous IIoT environment. To handle heterogeneous data in distributed IIoT networks, the FT-CID model exploits the FL and TL models to aggregate the attack data of the various RPL-IIoT network and ensure the design of customised edge IDS models, respectively. Federated learning consists of two learning models: server-side and edge-side models. During network initialisation, the central security server trains its global model on a publicly available source dataset and initialises the global learning model.

The edge devices execute the distributed local learning models for the IIoT network dataset. With the assistance of TL, the edge devices transfer local models in terms of model parameters, such as the number of hidden layers, hidden neurons, and activation functions; logits in terms of probability values (the vector of raw predictions); and weights from the learning model to the central security server. Instead of sending all data to the central server for global model generation, the proposed FT-CID model transfers only the logit outputs of the specified CNN layer of the local learning models and sends the obtained local information as knowledge to the server. Moreover, the proposed model optimises the parameters of the local learning model in terms of the number of iterations based on the gradient obtained from the local learning results. During aggregation in the FT-CID model, the server aligns the local parameters in the local model by employing the degree of importance of factors with the attention mechanism and applies FedAVG [36] to the received models of different edges to generate a global model. The FedAVG method is responsible for updating the model multiple times before sending the learning model back to the central or global server for aggregation. The FL model ensures that the central server aggregates the parameters from the sensors to update the global model in each communication round. With the centralised characteristics of the FedAVG model, there is a need for a very high number of communication rounds between the sensor and the server in order to collect parameters of the sensors and distribute the parameters to each sensor. Finally, the global parameters are sent back to the edge of the IIoT network to optimise the local learning models and detect intrusions.

#### 4.3.1. Distributed Local Model Training with Parameter Transfer 

The FT-CID model first builds the local learning model at the edges of the heterogeneous IIoT network to train the IIoT data samples with the optimal feature set. In the FT-CID model, the IDS located at the edge of the IIoT network exploits the CNN model to perform the local learning process on the data samples of the IIoT network. With the advantage of a learning model with minimal parameters in the CNN, the proposed FT-CID adopts the CNN deep learning model for intrusion detection. The network traffic of the optimal features of the HIIoT is provided as input to the CNN. In the FT-CID, the CNN structure comprises the following layers: input, convolution, pooling, flattening, dropout, full connection, softmax, and output. According to the concept behind the TL model, layers are frozen during learning, whereas the flattening, dropout, and full connection layers are used to transfer parameters, along with the logits and weights. To retain the optimal set of features, the FT-CID model freezes the convolution and pooling layers in the CNN model. Furthermore, the softmax layer is used to optimise the local learning process.

In the FT-CID model, the local learning model results in output in the form of logits. The FT-CID builds CNN local learning models (CNN1) using the datasets (D1). Furthermore, the updated local learning outputs of the fully connected layers with weights are fed into the global learning process as inputs through TL. The FT-CID model optimises the parameters of the local learning model with the optimal number of iterations based on the gradient of the learning results. The optimisation of the local learning model relies on weighted aggregation-based globally shared parameters and attention mechanisms. By iteratively monitoring the gradient of each local and global learning model, the FT-CID model customises the local learning model with the optimal number of iterations. Moreover, it transfers the parameters of the local learning model according to the degree of importance of each model, in addition to transferring the logits and weights to generate the global model. 

#### 4.3.2. Global Model Generation with Relational Knowledge Transfer

Following local model learning, it is crucial to transfer or update the specified local parameters to the server in order to build a global shared model in the FTL-based IDS. The TL model aims to transfer the local parameters generated with gradient-based weights from the edges to the central server. The TL partially retrains the predefined learning of the central server, which limits the complexity of the algorithm and increases the accuracy of global model generation. In the FT-CID model, the local learning models comprise local parameters with logits between 0 and 1, which are considered relational knowledge values. In the local learning model, parameter transfer involves the transfer of the learning model parameters, logits, and weights. The local learning model parameters and logits mainly refer to the patterns learned from the known and unknown types of intrusions of every RPL-IIoT domain, and the weights denote the importance of the local learning model parameter for the results. The logit of each local model is updated to the security sensor server using a TL algorithm. The TL process with parameter transfer and relational knowledge transfer during the local-to-global updating and global-to-local optimisation processes is shown in Figure 2 and Figure 3. After global parameter modelling, the FT-CID model carries the global intrusion patterns in terms of new logits and weights to the edges for local learning model optimisation with the assistance of the TL model.

The FT-CID model builds the global shared model with the weighted average of the local learning models using FedAVG for the heterogeneous IIoT edges. The FedAVG computes the differing importance of weight degree values for each local model. Hence, the edge of the IIoT network has a different importance value in the FT-CID model based on the impact of the local learning model parameters on the global model and the gradient. The FedAVG then computes the standard importance degree for each edge of the IIoT network based on the local model contribution parameter weights and gradients and thus optimises the updating of the global shared model. The local model receives high weights when the contribution is high with the assistance of attention weight modelling. As a result, the FT-CID server not only aggregates the local parameters but also considers the weights of the local learning model to accelerate global model convergence. The central server receives local model updates from ‘n’ different edges through TL, and it starts to retrain its predefined model based on the significance of the local models. For instance, the local models of different edges are denoted as LM= {CNN1}, the corresponding weights are denoted as WLM={W1}, and the global model on the centralised server is modelled using Equation (2) across ‘M’ number of IIoT networks.

(2) $$W_{\mathrm{GM}}=\overset{M}{\underset{E|\mathrm{LM}=1}{\sum }}\left[{\left|N\right|}_{\mathrm{LM}}{\;*\;h}_{E}\left(i-1\right){\;*\;W}_{\mathrm{LM}}\right]$$

where ‘N’ refers to the total number of samples in each local learning model or IIoT network, and $W_{\mathrm{GM}}$ refers to the weight of the global learning model. In the FT-CID model, the weight of the global model relies on the weight of the local learning model, which is determined by the contribution level of each local model to global level optimisation through attention weight modelling. By applying Equation (3), the FT-CID model measures the influence of each local learning model to the global learning model.

(3) $$d\left(W_{\mathrm{GM}}\left(i\right),W_{\mathrm{LM}}\left(i\right)\right)=\sqrt{\overset{n}{\underset{i=1}{\sum }}{\left({\left(W_{\mathrm{GM}}\right)}_{i}-{\left(W_{\mathrm{LM}}\right)}_{i}\right)}^{2}}$$

where $d\left(W_{\mathrm{GM}},W_{\mathrm{LM}}\right)$ refers to the difference between the weights of the global shared model and each local model during the ith iteration, where ‘i’ ranges from 1 to n. Furthermore, ‘d’ is used to estimate the contribution of the local model parameters of each sensor to global model optimisation. FL requires multiple iterations to generate a global model. Hence, the FT-CID model optimises the iterations with the help of attention-mechanism-based global model updating.

(4) $$h_{E}\left(i\right)=\frac{M\times w_{E}\left(i\right)}{\sum_{E=1}^{M}w_{E}\left(i\right)}$$

The FT-CID model minimises the communication rounds for convergence with the influence of the attention mechanism using Equation (4), where ‘M’ denotes the number of local models or network environments, and $w_{E}\left(i\right)$ refers to the weight of the local learning model parameters from the environment (E) for each ith iteration. In the FT-CID model, the gradient and dimensional difference-based attention mechanism limits the number of communication rounds and enhances the convergence speed without degrading the aggregation efficiency. The global shared model is generated with new logits and sent to each edge IDS to customise the local model.

(5) $$w_{E}\left(i\right)=f_{\mathrm{sigmoid}}\left[\frac{d\left(W_{\mathrm{GM}}\left(i\right),W_{\mathrm{LM}}\left(i\right)\right)}{\sigma_{E}\left(i\right)}\right]$$

The FT-CID model formulates Equation (5) to compute the gradient ($\sigma_{E}\left(i\right)$) and dimensional difference-based normalised result to update the attention weight of the learning model at each ith iteration. In Equation (5), the gradient refers to the results of the local learning model on the local datasets, which assist in aggregating the local model parameters within the optimal number of iterations until reaching the optimal convergence. 

#### 4.3.3. SGD-TL-Based Local Model Optimisation

The FT-CID model uses global model knowledge and logits to optimise the local learning model with partial retraining on the local learning model. During local model optimisation, the new logits comprise the global knowledge of unknown attacks of the various RPL-IIoT networks. Moreover, the FT-CID model selects the relevant source domain to build the pretrained FTL model with the aid of the SGD method. The FT-CID inputs the new logits to the softmax layer of each local CNN and partially retrains the local learning model based on global knowledge, resulting in high intrusion detection accuracy. The TL model of the FT-CID between the server and sensor edge of the IIoT network utilises the SGD model to optimise the local learning model. To address the constraints of bandwidth-limited networks, the FT-CID aims to apply the standard optimisation methods of the SGD to the local models rather than using the SGD iterations to update the global model. In the FT-CID model, sensor updating relies on the results from the multiple SGD iterations on its local datasets and then establishes communication with the server with reference to the local models, with the intention of reducing the number of communication rounds. SGD is an iterative method that optimises the local learning model based on the measure of variance between the server and the specified edge. The variance ($D_{V}$) reflects the difference between the source and target domain datasets in the concept of TL, in which the data pretrained on the server refer to the source domain, and the local data on the IIoT network refer to the target domain. In Equation (6), ${\mathrm{Lg}}_{s}$ and ${\mathrm{IIoT}}_{T}\left(E\right)$ denote the new logits in the Sth source domain and local data in the Tth target domain of the IIoT network, respectively. Finally, the FT-ID appends the distance value in the computation of the loss function of RPL IDS learning ($L_{\mathrm{LIDS}}$) to build the customised learning model for the IDS.

(6) $$L_{\mathrm{LIDS}}\left(E\right)=L\left({\mathrm{LM}}_{E}\right)+{{\lambda D}^{2}}_{V}\left({\mathrm{Lg}}_{S},{\mathrm{IIoT}}_{T}\left(E\right)\right)$$

The FT-CID model customises each local learning model for the IIoT network based on the loss function or gradient of the learning model during the adaptation of the new logits of the global learning model in the local learning model. In Equation (6), ‘λ’ balances the task of intrusion detection between the distance of the source and the target data, and $L\left({\mathrm{LM}}_{E}\right)$ refers to the loss of the individual Eth local learning model, which is used to update the loss value of the learning model. The FT-CID can then optimise the learning process of the local CNN of the IIoT network with the relevant global knowledge. The learning process of the FTL-enabled IDS is outlined in the process flow diagram shown in Box 1.

Box 1 **FTL-assisted RPL IDS learning.**

**//FTL-assisted RPL IDS Learning Process//**

Input: Local parameters of IIoT network and global shared knowledge

Output: Efficient Learning

The Edge IDS do {

Collects data samples from each RPL-IIoT network;

Performs CNN local learning;

Optimizes iterations with attention

Obtains the local parameters of a fully connected CNN layer;

Initiates Transfer Learning Process;

TL {

  Transfers the relational knowledge from edge to server;

  Server do {

    Estimates degree of importance of each IIoT local parameter;

    Aggregates the data using Equations (2) and (3);

    Generates attention weight of global model using Equations (4) and (5)

    Optimises iterations with attention mechanism;

    Generates a global shared model with new logits;

    Sends back to the edges through transfer learning;

    }

  Transfers the new logits to the softmax of local edge IDS;

  Optimizes the local learning process using Equation (6);

  }}

In the subsequent CNN local model optimisation, the FT-ID model initiates the intrusion detection task for the corresponding local datasets in the heterogeneous IIoT networks. By learning the patterns of the upcoming testing traffic data with the customised local learning model, the FT-CID model first classifies the data as normal and abnormal samples as intrusions. By implicitly exploiting the global attack knowledge in customised IDS learning, the proposed FT-CID model detects and classifies the intrusions over a heterogeneous IIoT environment. With the potential advantage of the global knowledge of various unknown attacks on different RPL-IIoTs, the FT-CID model significantly improves the quality of intrusion detection without compromising the RPL network performance.

## 5. Experimental Evaluation

To demonstrate the performance improvement of the proposed FT-CID model, the experimental model evaluates the proposed FT-CID model through the comparison of baseline models of different learning algorithms based on an IDS. It selects the baseline models, logistic regression (LR), multilayer perceptron (MLP), and CNN-based RPL-IIoT IDS due to the lack of existing research work in the area of FTL-based RPL-IIoT IDSs.

### 5.1. Experimental Setup

To implement an RPL-IIoT intrusion detection algorithm, the experimental model conducts experiments using the Python programming language. The FT-CID model runs on Ubuntu 18.04 LTS with an Intel i5 3.00GHZ CPU and 16 GB memory. The CNN-based FTL-IDS model is implemented using an FL model with the Keras API. The experimental model demonstrates the intrusion detection process with an AMI IIoT network. The experimental model implements the classification algorithms in a centralised and federated environment setup. The centralised environment setup refers to the training and decision making by the local learning model on the local IIoT network, whereas the federated environment setup refers to the training and collaborative decision making by the local and global learning models on the local IIoT networks. The centralised setup restricts several of the learning models trained on the entire heterogeneous RPL-IIoT dataset. The experimental model conducts experiments on the centralised setup for the LR, MLP, and CNN. In the centralised setup, the aggregator function is the arithmetic mean of the prediction probabilities of each learning model on the heterogeneous RPL-IIoT network (AMI). Moreover, the experimental model runs the CNN algorithm in the federated setup associated with the procedures involved in the FT-CID model.

### 5.2. Dataset Description

To evaluate RPL-IIoT intrusion detection in heterogeneous IIoT networks, the experimental model generates an RPL-IIoT dataset with normal and abnormal traffic for an IIoT network using Cooja Simulator. The dataset generation process is for a heterogeneous IIoT network (i.e., AMI) with different samples and the same feature space. With the support of Contiki-NG, the RPL-IIoT dataset samples comprise intrusion and normal samples; the intrusions involve rank, version, hello flood, DoS, blackhole, Sybil, selective forwarding, sinkhole, theft, and wormhole attacks. 

### 5.3. Performance Metrics

To validate the performance of the FT-CID model, the experimental model employs the widely used evaluation metrics described below. 

Precision: the ratio of the total number of samples correctly classified as intrusions relative to the total number of samples detected as intrusions expressed as a percentage.

$$\mathrm{Precision}=\left[\frac{\mathrm{True}\;\mathrm{Positive}}{\mathrm{True}\;\mathrm{Positive}+\mathrm{False}\;\mathrm{Positive}}\right]$$

Recall: the ratio of the total number of samples correctly classified as intrusions relative to the total number of actual intrusions expressed as a percentage.

$$\mathrm{call}=\left[\frac{\mathrm{True}\;\mathrm{Positive}}{\mathrm{True}\;\mathrm{Positive}+\mathrm{False}\;\mathrm{Negative}}\right]\;$$

F-measure: the harmonic mean of precision and recall expressed as a percentage.

$$F-\mathrm{measure}=2\times \;\frac{\mathrm{Precision}\times \mathrm{Recall}}{\mathrm{Precision}+\mathrm{Recall}}$$

Intrusion detection accuracy: the overall accuracy of correctly identified normal and abnormal traffic samples expressed as a percentage.

$$\;\mathrm{Accuracy}=\left[\frac{\mathrm{True}\;\mathrm{Positive}+\mathrm{True}\;\mathrm{Negative}}{\mathrm{True}\;\mathrm{Positive}+\mathrm{True}\;\mathrm{Negative}+\mathrm{False}\;\mathrm{Positive}+\mathrm{False}\;\mathrm{Negative}\;}\right]$$

False-Positive Rate: the ratio of legitimate samples incorrectly classified as intrusions relative to intrusions incorrectly classified in the corresponding attack category expressed as a percentage.

$$\;\mathrm{False}\;\mathrm{Positive}\;\mathrm{Rate}=\left[\frac{\mathrm{False}\;\mathrm{Positive}}{\mathrm{False}\;\mathrm{Positive}+\mathrm{True}\;\mathrm{Negative}\;}\right]$$

where:

- True positive (TP) = number of samples correctly detected as intrusion samples;
- True negative (TN) = number of samples correctly detected as normal samples;
- False positive (FP) = number of samples incorrectly detected as intrusion samples;
- False negative (FN) = number of samples incorrectly detected as normal samples.

## 6. Results and Discussion

To illustrate the performance of the proposed FT-CID model against the baseline IDS models, the experimental model evaluates the RPL-IIoT AMI dataset by varying the scenarios of the training data size and the number of communication rounds. To validate the results, we compared our FT-CID model with different algorithms in terms of accuracy, such as MLP, LR, and CNN, using the centralised setup to show significant improvements in intrusion detection accuracy. In this section, we also consider the influence of the communication rounds on intrusion detection performance.

### 6.1. Training Data Size vs. Precision 

**Figure 4:**
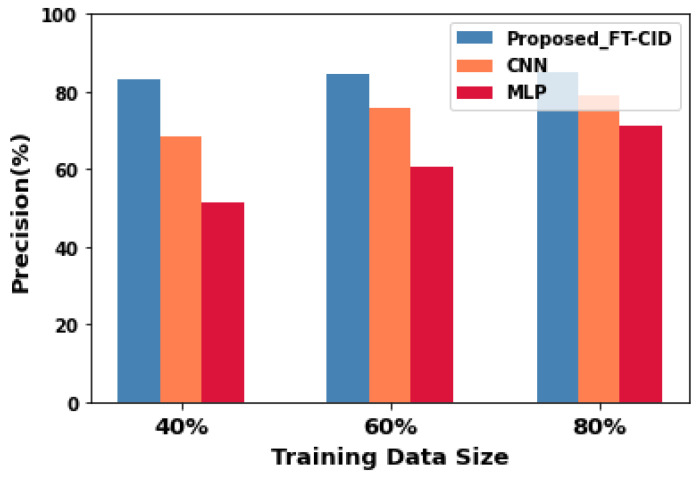
Precision performance for FT-CID, CNN, and MLP.

Figure 4 illustrates the effect of a varying number of training samples on the precision performance of the FL setup of the proposed FT-CID model by comparing the CNN and MLP algorithms in the centralised setup with the average score. The performance of the intrusion detection model improves with increased size of training samples. Precision performance is obtained for 20 communication rounds in the AMI-associated federated environment. The proposed FT-CID achieves a precision value of 85.18%, and the precision values of the CNN and MLP models are 78.92% and 71.09%, respectively. As the size of the training data increases, the precision of both the proposed intrusion detection model and the baseline algorithm-based intrusion detection model increases. Compared with the CNN in the centralised setup, the CNN model in the proposed federated setup shows a 14.33 percentage-point improvement in precision, even with only 40% of the local learning model, with the assistance of the transfer learning model associated with parameter transfer and relational knowledge transfer. By inherently analysing the multiple sets of training data, the proposed intrusion detection model learns the attack patterns from the heterogeneous IIoT network by updating the global model and optimising the local learning model with the assistance of the TL model associated with parameter transfer and relational knowledge transfer. By analysing the multiple attack patterns with the global and local knowledge, the FT-CID model shows greater accuracy in distinguishing the intrusion samples from the normal samples and has higher precision than the existing CNN and MLP models. Moreover, the FT-CID model customises the learning model based on the gradient of the local learning model and the knowledge from the global model, which tends to improve the performance of the comparative models. As a result, the customised modelling of the IIoT network tends to yield better TPs in the IDS, even when there is a lack of available intrusion patterns in the training data of the local IIoT network. Table 1 presents the numerical points of Figure 4.

### 6.2. Training Data Size vs. Recall

**Figure 5:**
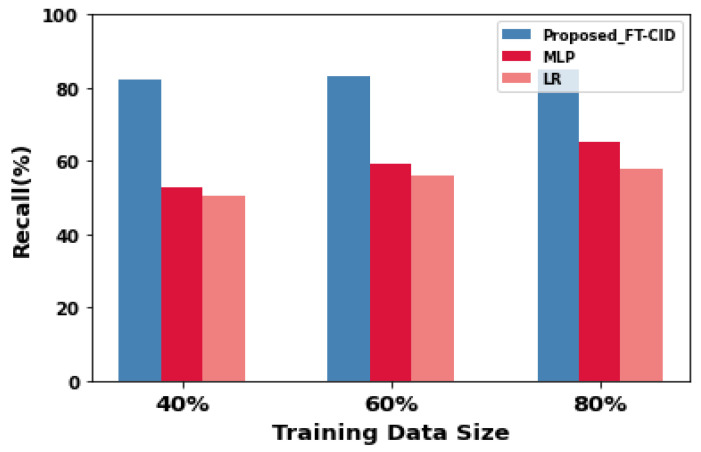
Recall performance for FT-CID, MLP, and LR.

As shown in Figure 5 and Table 2, the influence of training data size on recall performance is significantly higher in the RPL IDS. The FT-CID achieves higher recall performance for all three training data sizes in the federated setup (40%, 60%, and 80%) and for 20 communication rounds in the AMI heterogeneous IIoT network. MLP is ranked second, and LR recall is third—7.45 percentage points lower than the MLP and 27.03 percentage points lower than the FT-CID. The proposed FT-CID model improves intrusion detection performance, particularly in small training samples. Figure 5 shows that the recall obtained by the proposed FT-CID model is better than that obtained by the MLP and LR models for IIoT dataset for all the training data sizes. According to modelling of the federated environment for heterogeneous IIoT applications, the performance of the FT-CID is better than that of the ML algorithms on the MLP and LR on the IIoT dataset. Moreover, the FT-CID yields 23.7 and 27.9 percentage-points higher recall than the baseline MLP and LR models, respectively, with 60% of training data due to the advantage of gaining knowledge from other IIoT datasets, as well as the source dataset, of the attack patterns during global model training and updating. 

### 6.3. Number of Communication Rounds vs. F-Measure

**Figure 6:**
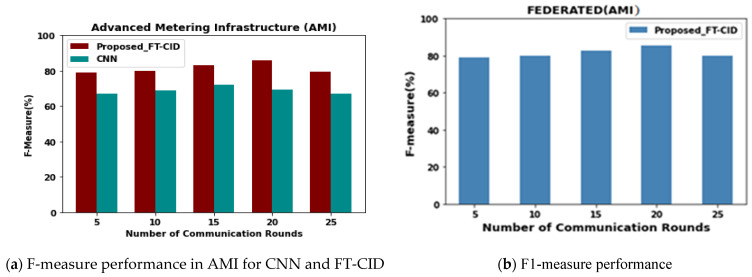
F-measure performance.

Figure 6 compares the F-measure of the proposed FT-CID model with that of the neural network models in local IIoT environments (i.e., AMI). Figure 6b shows the F-measure of the proposed intrusion detection model, the FT-CID, with various communication rounds (5, 10, 15, 20, and 25) for 80% of the training data size. Based on analysis of the results shown in Figure 6a,b, it can be determined that the centralised and federated intrusion detection models tend to converge after a certain number of communication rounds. Thus, the proposed intrusion detection model has a 16.83% higher F-measure than the baseline of the CNN in the independent AMI IIoT environment due to the advantage of collaboratively utilising the knowledge from the heterogeneous IIoT environments for 20 communication rounds. As the number of communication rounds increases from 5 to 25, the F-measure performance of each intrusion detection model increases and gradually stabilises, and the performance is reduced after reaching a sufficiently large number of iterations or communication rounds. Moreover, Figure 6b shows the F-measure performance of the FT-CID model with the average AMI heterogeneous IIoT environment score for the various numbers of communication rounds. The data values from the implementation F-measure results are provided in Table 3.

### 6.4. Intrusion Detection Accuracy

**Figure 7:**
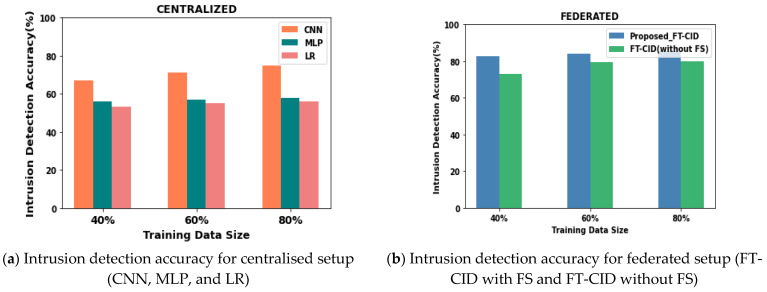
Intrusion detection accuracy performance.

Compared with the CNN and MLP neural network models, the FT-CID significantly improves the detection accuracy when tested in a heterogeneous IIoT environment. Furthermore, the experimental results show that the neural network models have stronger intrusion detection performance than the neural network and ML, MLP, and LR algorithms. The FT-CID obtains the best detection performance, even when there is a minimum number of training data sizes. Figure 7 and Table 4 show the implementation results of the performance of the centralised and federated intrusion detection models in terms of accuracy under scenarios of different percentages of training data size and for 20 communication rounds. Figure 7b shows that the proposed FT-CID model integrated IGWO-based feature selection (FS) outperforms the FT-CID model without FS, with 5.49% higher intrusion detection accuracy when there is 80% training data. As shown in Figure 7, the performance accomplished by the FT-CID is better than that of the neural network and ML models of the CNN, MLP, and LR in centralised settings. By applying the IGWO algorithm, the FT-CID model selects the optimal set of features from the raw IIoT dataset and thus ensures improved detection accuracy compared to the detection of intrusions by the MLP and LR models without FS of 22.7% and 24.69% 60% of training data, respectively. 

### 6.5. Training Data Size vs. False-Positive Rate

**Figure 8:**
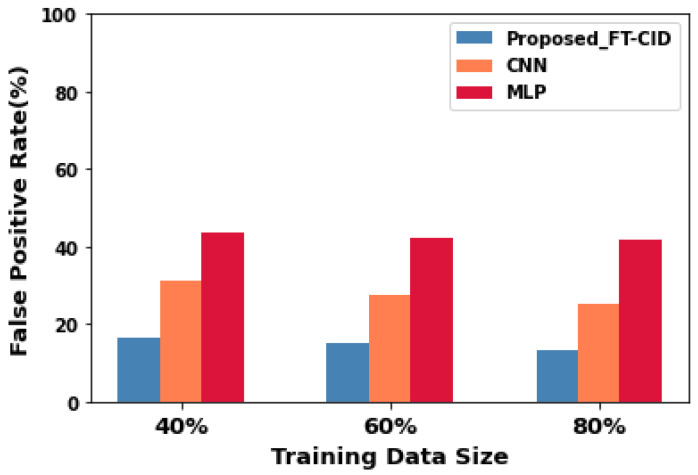
False-positive rate performance for FT-CID, CNN, and MLP.

Figure 8 shows the FP rate of the proposed FT-CID and baseline CNN and MLP models under different training data sizes and 20 communication rounds in AMI IIoT environments. As the size of the training data increases, the FP rate of the FT-CID, CNN, and MLP decreases. When the training data size is 80%, the FP rate of the FT-CID is still below 16%, but the FP rates of the CNN and MLP are higher, at 24.98% and 41.76%, respectively. The principle behind the above phenomenon is that the proposed FT-CID learns the related attack patterns from a similar intrusion detection task using FL and TL methods. Moreover, the FT-CID optimises the local learning model with the assistance of SGD and relevant knowledge learned from the global model. Consequently, the FT-CID improves the intrusion detection performance with a reduced number of FPs in the target task and maintains higher intrusion detection accuracy even with a small training data size. The numerical points obtained from the experiments for the FP rate are shown in Table 5.

## 7. Conclusions

In this paper, we presented the design of a proposed customised IDS for a heterogeneous RPL-IIoT network with the assistance of a deep FTL model. First, the proposed FT-CID model removes the noise and filters irrelevant features from the simulated RPL-IIoT dataset by modelling the IGWO algorithm. In the FT-CID model, the deep FTL algorithm relies heavily on the CNN algorithm to build local models based on the IIoT network, i.e., global model updating and local model optimisation. The logit output of each local model greatly influences the updating of the global model in the federated server. Thus, the logits in the global model assist in the optimisation of the local models in the sensors or the IIoT networks. In the FT-CID model, the TL model involves parameter transfer and relational knowledge transfer during global model updating and local model optimisation with the SGD model. Thus, the experimental results illustrate that the proposed FT-CID model yields 10.63% higher intrusion detection accuracy than the centralised CNN model in a heterogeneous IIoT network or AMI applications.

In future work, the data collected in this study will be analysed and processed to analyse the attack intention of intruders or correctly predict the intruder’s intrusion behaviour.

## Figures and Tables

**Table 1:** Comparative experimental precision results.

Training Data Size (%)	Precision (%)		
	Proposed FT-CID	CNN	MLP
**40**	82.97	68.64	51.25
**60**	84.32	75.63	60.83
**80**	85.18	78.92	71.09

**Table 2:** Comparative experimental recall results.

Training Data Size (%)	Recall (%)		
	Proposed FT-CID	MLP	LR
40	82.12	52.95	50.62
60	83.08	59.38	55.99
80	84.94	65.36	57.91

**Table 3:** Comparative experimental results of F-measure.

Number of Communication Rounds	F-Measure (%)		
	Centralised		Federated
	AMI		
	Proposed FT-CID	CNN	Proposed FT-CID
5	79.23	67.23	78.97
10	80.16	68.98	80.12
15	83.296	72.15	82.72
20	85.91	69.15	85.75
25	79.56	67.16	79.98

**Table 4:** Comparative experimental results of intrusion detection accuracy.

Training Data Size (%)	Intrusion Detection Accuracy (%)				
	Centralised			Federated	
	CNN	MLP	LR	Proposed FT-CID	Proposed FT-CID (without FS)
40	67.16	55.86	53.25	82.83	73.24
60	71.23	56.95	54.96	84.17	79.65
80	74.89	58.04	56.12	85.52	80.03

**Table 5:** Comparative experimental results of false-positive rate.

Training Data Size (%)	False-Positive Rate (%)		
	Proposed FT-CID	CNN	MLP
40	16.57	31.251	43.74
60	15.01	27.36	42.17
80	13.29	24.98	41.76

## Data Availability

Not Applicable.
